# Identifying Outstanding Transition-Metal-Alloy Heterogeneous Catalysts for the Oxygen Reduction and Evolution Reactions via Subgroup Discovery

**DOI:** 10.1007/s11244-021-01502-4

**Published:** 2021-09-02

**Authors:** Lucas Foppa, Luca M. Ghiringhelli

**Affiliations:** 1 The NOMAD Laboratory, Fritz-Haber-Institut der Max-Planck-Gesellschaft, Faradayweg 4-6, 14195 Berlin, Germany; 2 Humboldt-Universität zu Berlin, Zum Großen Windkanal 6, 12489 Berlin, Germany

**Keywords:** Artificial intelligence, Subgroup discovery, Symbolic inference, Supervised descriptive rule induction, Transition-metal surfaces

## Abstract

In order to estimate the reactivity of a large number of potentially complex heterogeneous catalysts while searching for novel and more efficient materials, physical as well as data-centric models have been developed for a faster evaluation of adsorption energies compared to first-principles calculations. However, *global* models designed to describe as many materials as possible might overlook the very few compounds that have the appropriate adsorption properties to be suitable for a given catalytic process. Here, the subgroup-discovery (SGD) *local* artificial-intelligence approach is used to identify the key descriptive parameters and constrains on their values, the so-called SG rules, which particularly describe transition-metal surfaces with outstanding adsorption properties for the oxygen-reduction and -evolution reactions. We start from a data set of 95 oxygen adsorption-energy values evaluated by density-functional-theory calculations for several monometallic surfaces along with 16 atomic, bulk and surface properties as candidate descriptive parameters. From this data set, SGD identifies constraints on the most relevant parameters describing materials and adsorption sites that (i) result in O adsorption energies within the Sabatier-optimal range required for the oxygen-reduction reaction and (ii) present the largest deviations from the linear-scaling relations between O and OH adsorption energies, which limit the catalyst performance in the oxygen-evolution reaction. The SG rules not only reflect the local underlying physicochemical phenomena that result in the desired adsorption properties, but also guide the challenging design of alloy catalysts.

**Supplementary Information:**

The online version contains supplementary material available at 10.1007/s11244-021-01502-4.

## Introduction

Among the multiple processes that govern heterogeneous catalysis [1–3], the bond-breaking and -forming reactions occurring on the catalyst surface, and, in particular, the associated (free-) energy barriers, play an important role in determining the reactivity of a given material. The energy barriers of surface reactions have been related to the adsorption energy of reactants, reaction intermediates or products via linear Brønsted–Evans–Polanyi relationships [4, 5]. Adsorption energies can be evaluated using *ab initio* methods, for instance via density-functional-theory (DFT) calculations. However, the explicit evaluation of adsorption energies by accurate first-principles methods for a large number of materials, desirable in the context of catalyst screening, becomes impractical when complex catalysts such as transition-metal alloys are considered. This is because these materials display a large number of surface sites that are possibly relevant in catalysis.

In order to efficiently explore a large number of possibly complex materials in the quest for novel catalysts, the scaling-relations approach [6], among other physical [7] or data-centric [8] models, have been used for the estimation of adsorption energies at lower computational effort compared to DFT. The scaling relations exploit the approximately linear relationships between adsorption energies of different surface species to reduce the number of explicit DFT calculations needed to investigate a certain catalytic process. Such linear models are designed to estimate adsorption energies for as many different materials and surface sites as possible. However, only very few of the investigated systems present the appropriate adsorption properties to be useful for a given catalytic process. Firstly, the adsorption energies of key reaction intermediates typically need to lie in a Sabatier-optimal range for the performance to be maximized [9–11]. Secondly, the adsorption energies of different species might need to be tuned independently for an optimal reactivity to be achieved [12]. This implies that deviations from the linear relationships between adsorption energies, which describe the trend for most of the materials, might be actually desirable [13]. In both these situations, the interesting materials and surface sites thus present *statistically exceptional* adsorption properties. This questions the suitability of using global models to screen for new catalysts.

Here, we apply the subgroup-discovery (SGD) artificial-intelligence *local* approach [14–19] to identify key descriptive parameters—and constraints on their values-, which are particularly associated to outstanding adsorption properties of transition-metal surfaces. In particular, we introduce a strategy to address target properties whose desired values lie in a specific range and use this approach to describe adsorption sites presenting Sabatier-optimal oxygen adsorption energies for the oxygen-reduction reaction (ORR) [20]. Additionally, we show how SGD can be used to describe data points that deviate the most from a given model, such as the linear-scaling relations between O and OH adsorption energies on different surface sites. Such scaling relations impose a limit for the optimization of oxygen-evolution reaction (OER) performance [21]. Thus, materials and adsorption sites deviating from the linear scaling are the interesting ones. The ORR and the OER are two crucial processes for energy conversion and storage.

## Subgroup-Discovery Approach

We start our analysis by introducing the SGD approach [14–18] to uncover complex patterns associated to outstanding local behavior by using data sets. This methodology has been recently applied to catalysis [22] as well as materials-science [18, 23] problems.

**Fig. 1:**
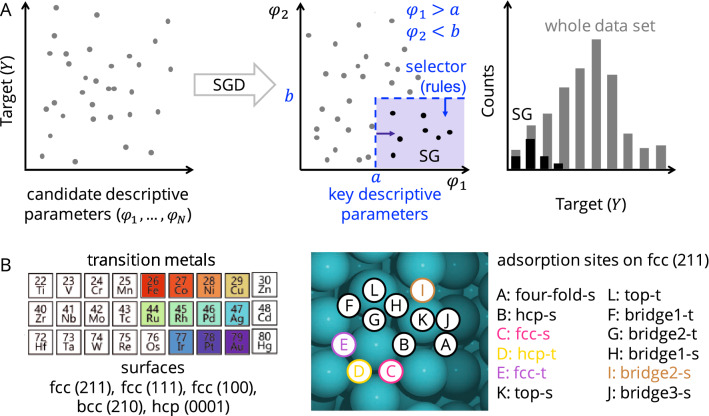
**A** illustration of the SGD approach for identifying key descriptive parameters and rules determining SGs with outstanding distribution of the target. The rules are constraints on the values of key descriptive parameters. The distribution of target values in the SG might be outstanding because it is, for instance, narrower than the distribution of the target values over the whole data set. **B** transition metals and surfaces considered in this work. We consider the face-centered cubic (fcc) structure for all metals except Fe, for which the body-centered cubic (bcc) structure and the (210) surface is considered. For Co, the (0001) surface of the hexagonal closed packed (hcp) structure is also included. The adsorption sites of the fcc(211) surface are also shown in detail on the right. This surface termination contains both terrace and step-edge-like sites, labelled “t” and “s” in the figure, respectively

The SGD method is based on an input data set, which we refer to as the *population* $P$ of data points, each of them associated to a different material or, in the case of this work, to a different surface site. For each of the data points, the value of a *target* of interest, $Y$, and the values of $N$ potentially relevant *candidate descriptive parameters*, denoted ${\varphi }_{1},{\varphi }_{2},\dots ,{\varphi }_{N}$, are known. The candidate descriptive parameters are structural or physicochemical parameters that possibly correlate with the target. Starting from such data set, SGD identifies subsets of data, hereafter *subgroups* (SGs), that present an outstanding distribution of the target values with respect to the whole data set (Fig. 1A). The so-called *quality function* $Q\left(P,SG\right)$ measures how outstanding a SG is compared to the whole data set. This function typically has the form

1 $$Q\left(P,SG\right)=\frac{s(SG)}{s(P)}* u\left(P,SG\right),$$

where the first term, the *coverage*, contains the ratio between the number of data points in the subgroup $s$(SG) and the total number of data points in the whole data set $s$(P). The coverage controls the subgroup size and prevents that very small SGs with little statistical significance are selected. The second term $u(P,SG)$, the *utility function*, measures the dissimilarity between the SG and the population. It can be chosen[18] depending on the scientific question of interest (vide *infra*).

The SGD algorithm consists in two steps. Firstly, combination of statements (hereafter *selectors*, $\sigma (\varphi )$) about the data are generated. The selectors are Boolean functions defined through conjunctions of propositions and have the form

2 $$\sigma (\varphi )\equiv {\pi }_{1}(\varphi )\wedge {\pi }_{2}(\varphi )\wedge \dots \wedge {\pi }_{p}(\varphi ),$$

where “$\wedge$” denotes the “and” operator and each proposition ${\pi }_{i}$ is, for instance, an inequality constraint on one of the descriptive parameters

3 $$ \pi _{i} \left( \varphi  \right) \equiv \varphi _{i}  \ge v_{i} \;{\text{or}}\;~\pi _{i} \left( \varphi  \right) \equiv \varphi _{i}  < v_{i} ,$$

for some constant ${v}_{i}$ to be determined during the analysis (see below). The *selectors* describe convex regions in the descriptive parameter space defining the SGs. Secondly, a Monte Carlo search algorithm is used to find SGs, defined by the selectors generated in the first step, that maximize the quality function. The most relevant SGs are those for which the quality function reaches the highest values. The selectors defining those SGs, and, more specifically, the propositions in the selectors, contain the key descriptive parameters associated to the underlying processes that exclusively govern the local behavior within the subsets (or SGs) of data points. The propositions entering the selectors can be thus seen as *rules* determining the outstanding SG behavior. Therefore, the SG is at the same time the subset of selected data and the selector, i.e., the rules that are used to obtain this selection. In fact, the SG rules are more relevant than the particular subset of selected (training) data. For candidate descriptive parameters that are continuous variables, ${v}_{i}$ (in Eq. 3) could assume any value within the ranges of variation of the descriptive parameters in the training data set. Thus, a large number of propositions could be, in principle, constructed using many different ${v}_{i}$ values. However, the SG search becomes computationally inefficient as the number of propositions increases. For this reason, only a finite set of meaningful ${v}_{i}$ values is taken into account in the SGD approach. These meaningful values are determined, for each candidate descriptive parameter, by *k*-means clustering using the input data. In order words, the clustering approach is used to select the *k* bins according to which the histograms associated to the distribution of each descriptive parameter are partitioned. Propositions are then formed, based on each of the resulting bins. In this work, we used 10 clusters. Further SGD details are available in Electronic Supporting Information, ESI.

## Data Set of Adsorption Energies and Candidate Descriptive Parameters

We analyze a data set containing 95 oxygen (atomic O) adsorption energies, which were calculated with DFT using the van der Waals-corrected BEEF-vdW exchange–correlation functional in previous publications. [8, 24] Eleven transition metals and several adsorption sites of different surfaces for which (meta)stable oxygen adsorption is observed were included in our analysis (Fig. 1B). We note that high- as well as low-coordinated metal sites are present in the chosen metal surfaces. In particular, the fcc(211) surface was considered because it contains both terrace and step-edge-like sites. By including sites with different coordination in our analysis, we take into account that the adsorption properties are sensitive to the surface structure and that either high- or low-coordinated sites might be relevant for catalysis. The oxygen adsorption energy is defined (using the convention in [11]) as

4 $${E}_{\mathrm{ads}}^{\mathrm{O}}={E}_{\mathrm{surf},\mathrm{clean}}+0.5 {E}_{{\mathrm{O}}_{2}(\mathrm{g})}-{E}_{\mathrm{surf},\mathrm{ads}},$$

where ${E}_{{\mathrm{O}}_{2}(\mathrm{g})}$, ${E}_{\mathrm{surf},\mathrm{clean}}$ and ${E}_{\mathrm{surf},\mathrm{ads}}$ are the total energies of the O_2_ gas-phase molecule, clean surface, and surface containing the O adsorbate, respectively. Positive oxygen adsorption-energy values correspond, therefore, to favorable adsorption with respect to the gas-phase molecule.

An important aspect in SGD is the choice of candidate descriptive parameters. Following reference [8], we use, as candidate descriptive parameters, the atomic, bulk, and clean surface properties shown in Table 1. The atomic parameters are properties that only depend on the element. The bulk, surface and site parameters are related to the geometry and the electronic structure of either bulk metals, or their surfaces and adsorption sites. The surface- and surface-site-related descriptive parameters were evaluated on (relaxed) clean surfaces, i.e., without the presence of the adsorbed species, in reference [8]. The surface-site parameters were calculated as averages over the metal atoms that compose the site ensemble. In total, 16 parameters uniquely characterizing each material and surface site are used. We note that the candidate descriptive parameter set includes properties proposed to describe overall trends in adsorption energies such as the *d*-band center (${\epsilon }_{d}$) [7] or coordination numbers (CN) [29] as well as many other, potentially relevant, parameters.

**Table 1:** Candidate descriptive parameters used for the SGD of outstanding transition-metal catalysts

Type		Description	Refs.
Atomic	$\mathrm{PE}$	Pauling electronegativity	[25]
	$\mathrm{IP}$	Ionization potential	[26]
	$\mathrm{EA}$	Electron affinity	[26]
Bulk	${\mathrm{bulk}}_{\mathrm{nnd}}$	Nearest-neighbor distance	[8]^a^
	${r}_{d}$	*d*-orbital radius	[27]
	${V}_{\mathrm{ad}}^{2}$	Coupling matrix element between the adsorbate states and the metal *d* states squared	[28]
Surface	$W$	Work function	[8]^a^
Surface Site	${\mathrm{site}}_{\mathrm{no}}$	Number of atoms in the ensemble	[8]^a^
	$\mathrm{CN}$	Coordination number	[8]^a^
	${\mathrm{site}}_{\mathrm{nnd}}$	Nearest-neighbor distance	[8]^a^
	${\epsilon }_{d}$	*d*-band center	[8]^a^
	${W}_{d}$	*d*-band width	[8]^a^
	${f}_{d}$	*d*-band filling	[8]^a^
	${f}_{sp}$	*sp*-band filling	[8]^a^
	${\mathrm{DOS}}_{d}$	Density of *d*-states at Fermi level	[8]^a^
	${\mathrm{DOS}}_{sp}$	Density of *sp*-states at Fermi level	[8]^a^

^a^As determined by DFT-BEEF-vdW

## Subgroups of Surface Sites with Optimal Range of Oxygen Adsorption Energies for the ORR

**Fig. 2:**
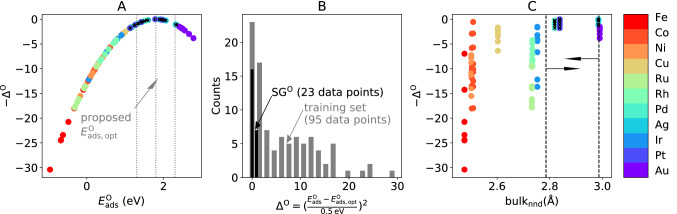
SGD of transition-metal catalysts presenting surface sites with an optimal range of oxygen adsorption energies. **A** visualization of the target quantity (${\Delta }^{\mathrm{O}}$), defined in Eq. 5, for the training data. ${\Delta }^{\mathrm{O}}$, which is unitless, is smaller than 1 in an interval of $\pm\, 0.5 \,\mathrm{eV}$ centered around the proposed optimal value of ${E}_{\mathrm{ads},\mathrm{opt}}^{\mathrm{O}}=1.8\, \mathrm{eV}$. **B** distribution of ${\Delta }^{\mathrm{O}}$ in the whole data set and in the identified SG. **C** SG rule, indicated by the dashed lines and by the arrows, on a identified key descriptive parameter: bulk nearest-neighbor distance (${\mathrm{bulk}}_{\mathrm{nnd}}$). The data points corresponding to the SG are marked with black crosses in **A** and **C**

To illustrate how SGD identifies the relevant descriptive parameters and the rules describing surface sites that bind a certain reaction intermediate with a specific binding strength, we start our analysis by identifying SGs of surface sites providing an optimal oxygen adsorption energy ${E}_{\mathrm{ads},\mathrm{opt}}^{\mathrm{O}}$ of 1.80 eV. Based on DFT-derived potential energy surfaces corresponding to the proposed main mechanisms of the ORR, adsorbed oxygen was identified as a key intermediate in this reaction and the oxygen adsorption-energy value of 1.80 eV was related to the highest activity over a series of transition-metal low-index (111) surfaces [11]. To take into account that a *range* of oxygen adsorption energies around 1.80 eV might result in catalysts that maximize the performance, we define, for our SGD analysis, a target that assumes small values in a given window around ${E}_{\mathrm{ads},\mathrm{opt}}^{\mathrm{O}}$ and rapidly increases outside such interval. Among several possible choices of functions that would reproduce this behavior, we use a quadratic expression and consider [1.30, 2.30 eV] window of optimal adsorption-energy values. Our SGD target is thus defined by

5 $${\Delta }^{\mathrm{O}}={\left(\frac{{E}_{\mathrm{ads}}^{\mathrm{O}}-{E}_{\mathrm{ads},\mathrm{opt}}^{\mathrm{O}}}{0.5 \mathrm{eV} }\right)}^{2},$$

where ${E}_{\mathrm{ads}}^{\mathrm{O}}$ is the oxygen adsorption energy for an arbitrary surface site. The distribution of ${\Delta }^{\mathrm{O}}$ over the training data set of 95 adsorption-energy values is shown in Fig. 2A and B. We are interested in SGs of data points for which ${\Delta }^{\mathrm{O}}$ assumes low values. As utility function, we use

6 $$u\left(P,SG\right)=\frac{std(SG)}{std(P)},$$

where $std(\mathrm{SG})$ and $std(\mathrm{P})$ are the standard deviation of the distributions of the target in the SG and in the whole data set, respectively. By using the ratio of standard deviations in the utility function, we favor the selection of SGs that present narrow distribution of values for the target.

Among the SGs that maximize the quality-function values, we identify a SG containing 23 data points, i.e., ca. 24% of the data set that presents a narrow distribution of target values relatively to the whole data set and is centered at the lowest target values (Fig. 2B, in black).

This SG contains the surface sites for which the oxygen adsorption energies are the closest to the proposed optimal value (Fig. 2A, in which the adsorption sites belonging to the SG are shown as black crosses). All considered adsorption sites of Pd, Ag and Pt surfaces are part of this SG. Pd and Pt are indeed known to be the best ORR catalysts among all metals included [20]. This SG is defined by the selector (Fig. 2C)

7 $${\sigma }^{\mathrm{O}}\equiv 2.786<{\mathrm{bulk}}_{\mathrm{nnd}}\le 2.987 \text{\AA} .$$

Therefore, the interatomic nearest-neighbours distance of the bulk materials is a key parameter associated to the optimal range of oxygen adsorption energies for the ORR. In particular, materials for which ${\mathrm{bulk}}_{\mathrm{nnd}}$ assumes an intermediate range of values, given by (7), present surface sites with the desired oxygen binding strength. We note that the SG rules do not necessarily reflect causality. The relevance of ${\mathrm{bulk}}_{\mathrm{nnd}}$ in (7), for instance, does not imply that the application of strain to reduce the ${\mathrm{bulk}}_{\mathrm{nnd}}$ in Au will improve the performance of this material. It might reflect, however, that both the equilibrium bulk interatomic distance and the oxygen adsorption are controlled by similar underlying bonding patterns.

The SG rule given by (7) is the simplest SG rule identified, which only depends on one descriptive parameter. Several different SG rules (Table S1) result, however, in the exact same subselection of (training) data points and thus in the same quality-function values compared to the SG defined by (7). For instance, the selector

8 $$\begin{gathered}   {\sigma} ^{{{\text{O}}^{\prime}}}  \equiv {\text{site}}_{{{\text{nnd}}}}  > 2.759\,{\text{\AA}}{\mkern 1mu}   \wedge ~\,{\text{PE}} \le 2.125, \hfill \\  \end{gathered}   $$

which depends on two descriptive parameters, also selects the adsorption sites of Pd, Ag and Pt. The presence of similar SGs defined by slightly different rules is due to the fact that different descriptive parameters encode similar physicochemical information. Indeed, some of the candidate descriptive parameters are correlated with each other. In particular, the Pearson correlation between ${\mathrm{bulk}}_{\mathrm{nnd}}$ and ${\mathrm{site}}_{\mathrm{nnd}}$ is equal to 0.99 and between ${\mathrm{bulk}}_{\mathrm{nnd}}$ and $\mathrm{PE}$ is 0.72 (Fig. S3). We note that correlations involving more than two descriptive parameters, which are not captured by the Pearson correlation scores, might be also present within the training data set. This is not a limitation for SGD, since it can identify different equivalent descriptive rules (with respect to a given input training data).

We have also used the SGD approach with a categorical target, which *classifies* surface sites presenting oxygen adsorption energy in the desired range and verified the dependence of the SG on the choice of interval size (see details in ESI). The resulting SG rules are similar to those shown in (7) and (8).

The evaluation of adsorption energies on surfaces of metal alloys is more resource-consuming for DFT compared to monometallic systems, as the number of possible metal combinations and surface sites grows significantly. Therefore, approaches indicating the most promising alloy compositions and surface sites to be investigated are desirable. To assess the transferability of the SG rules trained using monometallic systems to alloys, we used an additional alloy data set. This alloy data set contains information on (211) surfaces of 36 bimetallic alloys with 1:1 atomic ratio, evaluated by DFT in reference [8]. Such data set is split in two subsets. (i) The *test set* contains the 4 alloy compositions AgAu, AgPd, IrRu, and PtRh and, in total, 37 different adsorption sites. For such test set, the oxygen adsorption energies are explicitly calculated by DFT. This data set is used for evaluating the performance of the SG rules on the alloys. (ii) The *exploitation set* contains the descriptive parameters for 32 alloy compositions: AgIr, AgPt, AuCu, CuAg, CuIr, CuPd, CuPt, CuRh, CuRu, IrAu, IrPt, NiAg, NiAu, NiCu, NiIr, NiPd, NiPt, NiRh, NiRu, PdAu, PdIr, PdPt, PtAu, RhAg, RhAu, RhIr, RhPd, RuAg, RuAu, RuPd, RuPt, and RuRh. The exploitation set contains, in total, 323 different adsorption sites. This data set used for the screening of new promising alloys and surface sites. We note that the alloy atomic descriptive parameters are taken as the average between the atomic properties of the metal atoms which compose a given surface site.

**Fig. 3:**
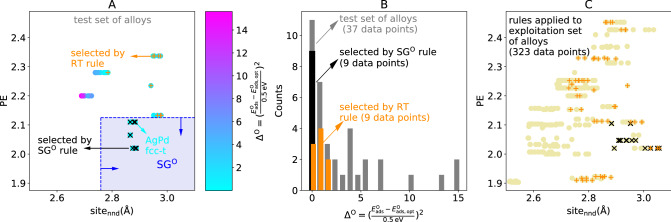
SG rules describing monometallic surface sites with optimal range of oxygen adsorption energies applied for the design of bimetallic alloys. **A** representation of the test set of alloy surface sites in the coordinates of the key descriptive parameters identified by the SG rule (8): ${\mathrm{site}}_{\mathrm{nnd}}$ and $\mathrm{PE}$. The data points are colored according to their DFT-calculated ${\Delta }^{\mathrm{O}}$ value. The data points selected by the SG rule (8) and by the regression tree rule (13) are shown in black and orange crosses, respectively. **B** distribution of DFT-calculated ${\Delta }^{\mathrm{O}}$ values in the test set of alloy surface sites (grey). The distributions of ${\Delta }^{\mathrm{O}}$ values over the data points selected by the SG rule (8) and the regression tree rule (13) are displayed in black and orange, respectively. **C** representation of the exploitation set of alloy surface sites in the coordinates ${\mathrm{site}}_{\mathrm{nnd}}$ and $\mathrm{PE}$. The data points selected by the SG rules shown in Table S1 (for the ${\Delta }^{\mathrm{O}}$ target) and by the regression tree rule (13) are shown in black and orange crosses, respectively

Figure 3A shows the surface sites of the test set of alloys in the coordinates of the two key descriptive parameters identified by the SG rule (8): ${\mathrm{site}}_{\mathrm{nnd}}$ and $\mathrm{PE}$. In this figure, the DFT-calculated ${\Delta }^{\mathrm{O}}$ values are indicated by the color code and the black crosses identify the alloy surface sites selected by the constraints in (8), the latter indicated by the blue dashed lines and arrows. In Fig. 3B, the distributions of ${\Delta }^{\mathrm{O}}$ values over the test set of alloys and over the data points selected by the SG rule are shown. Even though the SG rule misses two surface sites of the PtRh alloy (hcp-t-2 and fcc-t-2), which present relatively low ${\Delta }^{\mathrm{O}}$ of 0.11 and 0.14, respectively, it correctly indicates AgPd as an outstanding alloy.

Indeed, the ${\Delta }^{\mathrm{O}}$ values for the AgPd alloy lie in the range 0.00–0.48 and all AgPd surface sites are selected by the SG rule. Moreover, the SG rule did not select any alloy surface site with ${\Delta }^{\mathrm{O}}>0.48$. These results show that the SG rules trained only on monometallic systems have a good performance to describe alloys.

Next, we applied the SG rules to select surface sites of the exploitation set of alloys. In order to narrow down the selection, we use the additional constraint that the alloy surface sites of interest should simultaneously satisfy all the SG rules identified using the monometallic systems (rules shown in Table S1 for the ${\Delta }^{\mathrm{O}}$ target). For this reason, not all the data points falling in the region equivalent to the one shaded in Fig. 3A are marked with black crosses in Fig. 3C. The selection of alloys based on the SG rules results in the following alloys, identified as promising materials: AgIr, AgPt, and RhAg. While AgPt is obtained simply by combining two of the outstanding monometallic catalysts, the selection of AgIr and RhAg alloys indicates the potential of mixing Ag, which presents oxygen adsorption energy of ca. 2.0 eV with a second metal presenting oxygen adsorption energy slightly lower than 1.80 eV for achieving outstanding performance.

## Subgroups of Surface Sites Deviating from the Linear-Scaling Relations Between O and OH Adsorption Energies for the OER

The linear trends observed between adsorption energies of different surface species impose, in some reactions, a limit to the maximum performance that can be achieved. This is because the linear-scaling relations imply that the absorption of two related species cannot be tuned independently, limiting the possibilities for catalyst optimization. For instance, in the OER, the adsorption energies of the three key intermediates, O, OH, and OOH, are correlated [21] and the O adsorption energy needs be decreased with respect to OOH adsorption energy in order to decrease the limiting potential and thus maximize the performance [12]. To overcome this limitation imposed by the linear-scaling relations, an immense effort has been put into strategies to identify exceptional materials and adsorption sites that “break”, or deviate from, the scaling relations [13]. Most of the materials are typically well described by the linear-scaling relations. Thus, deviations from these linear models are the exceptions and local approaches might be more suitable for finding catalysts and surface sites that deviate from the scaling relations.

**Fig. 4:**
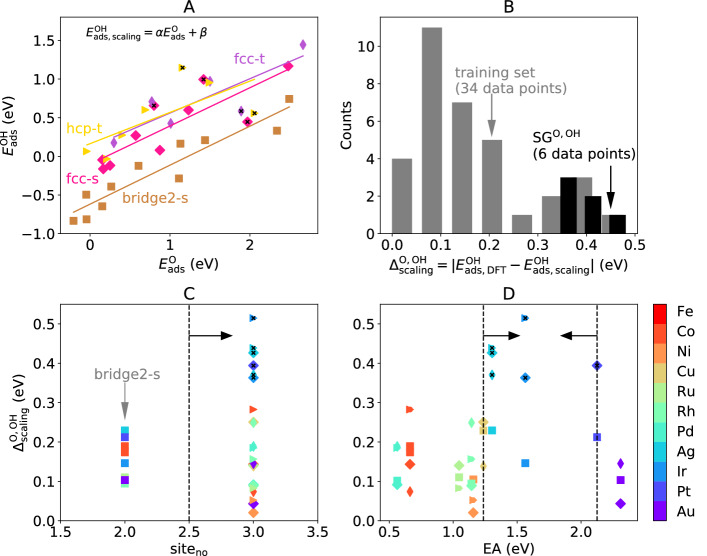
SGD of transition-metal catalysts and adsorption sites of fcc(211) surfaces that deviate from the linear-scaling relations. **A** scaling relations between oxygen (O) and hydroxyl (OH) species for different adsorption sites of the fcc(211) monometallic surfaces. **B** distribution of the target (${\Delta }_{\mathrm{scaling}}^{\mathrm{O},\mathrm{OH}}$) within the population and in the identified SG. **C** and **D** SG rules (indicated by the dashed lines and arrows) on the selected key descriptive parameters coordinates: number of atoms in the ensemble (${\mathrm{site}}_{\mathrm{no}}$) and electron affinity ($\mathrm{EA}$), respectively. The data points corresponding to the SG are marked with black crosses in **A**, **C** and **D**

To illustrate how the SGD approach can be used to address outstanding surface sites that deviate from linear-scaling relationships, we next search for SGs describing fcc(211) surface sites of monometallic surfaces providing high deviations from the scaling relations between atomic oxygen (O) and hydroxyl (OH). For this purpose, we first establish linear models for each adsorption site on which both O and OH present a (meta)stable adsorption: fcc-t, hcp-t, fcc-s and bridge2-s (show in colors in Fig. 4A). These models have the form

9 $${E}_{\mathrm{ads},\mathrm{scaling}}^{\mathrm{OH}}={\alpha E}_{\mathrm{ads},\mathrm{DFT}}^{\mathrm{O}}+\beta ,$$

where $\alpha$ and $\beta$ are fitted coefficients, different for each surface site. In total, 36 data points are used. The linear fits (Fig. 4A) evidence that most of the data points are well described by the scaling relation. Indeed, the deviations from the linear trend are typically lower than 0.20 eV (Fig. 4B). The bridge2-s surface site is in particular well captured by the linear model. We define the quantity

10 $${\Delta }_{\mathrm{scaling}}^{\mathrm{O},\mathrm{OH}}=\left|{E}_{\mathrm{ads},\mathrm{DFT}}^{\mathrm{OH}}-{E}_{\mathrm{ads},\mathrm{scaling}}^{\mathrm{OH}}\right|,$$

the absolute difference between the OH adsorption energy estimation by the scaling relation (${E}_{\mathrm{ads},\mathrm{scaling}}^{\mathrm{OH}}$) and the actual DFT-calculated value (${E}_{\mathrm{ads},\mathrm{DFT}}^{\mathrm{OH}}$) as target for the SGD approach. In this way, the interesting data points, i.e., the surface sites that are worst described by the linear trend, correspond to high values of ${\Delta }_{\mathrm{scaling}}^{\mathrm{O},\mathrm{OH}}$. Most of the observations in the data set correspond to low ${\Delta }_{\mathrm{scaling}}^{\mathrm{O},\mathrm{OH}}$ values (Fig. 4B). We are thus interested in SGs with an overall distribution of the target value as different as possible from the distribution of this quantity in the whole data set. This requirement can be introduced in the SGD by means of the following utility function:

11 $$u\left(P,SG\right)={D}_{\mathrm{cJS}}\left(P,SG\right).$$

In (11), ${D}_{\mathrm{cJS}}(P,SG)$ is the cumulative-distribution-function formulation [30] of the Jensen-Shannon divergence between the distribution of the target values in the SG and the distribution of the target values in the whole data set [30]. ${D}_{\mathrm{cJS}}$ measures the dissimilarity between two distributions. It assumes small values for similar distributions and increases as the distributions have different standard deviations and/or mean values (see further details in ESI). The candidate descriptive parameters shown in Table 1 are also used here, and only the monometallic systems are initially considered.

The SGD approach identifies a SG containing 6 data points, i.e., ca. 17% of the population, which is narrow and has relatively high target values with respet to the whole data set (Fig. 4B, in black). Indeed, this SG contains the surface sites deviating the most from the linear-scaling relations (Fig. 4A, in which the data points belonging to this SG are shown as black crosses). The sites fcc-s, fcc-t, and hcp-t of the Ag surface, the sites fcc-s, and hcp-t of the Ir surface and the fcc-s site of the Pt surface are part of this SG. Such SG is defined by the selector

12 $$\begin{gathered}   \sigma ^{{\text{O,OH}}}  \equiv \text{site}_{{\text{no}}}  > 2.5   \wedge 1.236\,\text{eV} \le \text{EA} \le 2.125~\text{eV}, \hfill \\  \end{gathered}  $$

as shown in Fig. 4C and D. Therefore, the number of atoms in the surface site ensemble (${\mathrm{site}}_{\mathrm{no}}$) and the electron affinity of the metal ($\mathrm{EA}$) are relevant parameters related to high ${\Delta }_{\mathrm{scaling}}^{\mathrm{O},\mathrm{OH}}$. The constrain on ${\mathrm{site}}_{\mathrm{no}}$ excludes the bridge2-s sites from the SG and shows that surface sites composed by more than two atoms, on which the adsorbate can be more highly-coordinated, are more prone to deviate from the linear trend. The conditions on $\mathrm{EA}$, in turn, shows that this outstanding behavior is limited to only some of the metals, and this is encoded in this element-dependent (atomic) parameter.

**Fig. 5:**
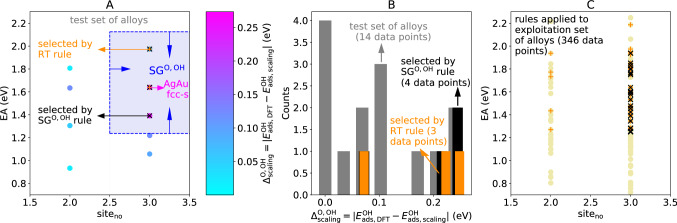
SG rules describing monometallic surface sites deviating from scaling relations applied for the design of bimetallic alloys. **A** representation of the test set of alloy surface sites in the coordinates of the key descriptive parameters identified by the SG rule (12): ${\mathrm{site}}_{\mathrm{no}}$ and $\mathrm{EA}$. The data points are colored according to their DFT-calculated ${\Delta }_{\mathrm{scaling}}^{\mathrm{O},\mathrm{OH}}$ value. The data points selected by the SG rule (12) and by the regression tree rule (14) are shown in black and orange crosses, respectively. **B** distribution of DFT-calculated ${\Delta }_{\mathrm{scaling}}^{\mathrm{O},\mathrm{OH}}$ values in the test set of alloy surface sites (grey). The distributions of ${\Delta }_{\mathrm{scaling}}^{\mathrm{O},\mathrm{OH}}$ values over the data points selected by the SG rule (12) and the regression tree rule (14) are displayed in black and orange, respectively. **C** representation of the exploitation set of alloy surface sites in the coordinates ${\mathrm{site}}_{\mathrm{no}}$ and $\mathrm{EA}$. The data points selected by the SG rules shown in Table S1 (for the ${\Delta }_{\mathrm{scaling}}^{\mathrm{O},\mathrm{OH}}$ target) and by the regression tree rule (14) are shown in black and orange crosses, respectively

We then evaluated the performance of the rules defining the SGs of surface sites deviating from the linear-scaling relations (12), derived based on monometallic systems, on the test set of alloys (Fig. 5A and B). The SG rules indicate the alloy surface sites AgAu fcc-s-1, AgAu fcc-s-2, AgAu hcp-t-2 and IrRu fcc-s-1 as those deviating the most from the scaling relations. Even though the AgAu fcc-s-2 presents ${\Delta }_{\mathrm{scaling}}^{\mathrm{O},\mathrm{OH}}=0.072 \mathrm{eV}$ and it is thus incorrectly selected by the SG rule, the AgAu fcc-s-1, AgAu hcp-t-2 and IrRu fcc-s-1 sites do correspond to the alloy surface sites with highest calculated ${\Delta }_{\mathrm{scaling}}^{\mathrm{O},\mathrm{OH}}$ values (0.27, 0.22, 0.25 eV, respectively). These results show that the SG rules derived based on monometallic systems have a reasonable performance for the alloys. By applying the SG rules shown in Table S1 for the ${\Delta }_{\mathrm{scaling}}^{\mathrm{O},\mathrm{OH}}$ target to the exploitation set of alloys (Fig. 5C), several fcc and hcp sites of the alloys AgIr, AgPt, AuCu, CuAg, CuIr, CuPt, IrPt, NiAg, NiPt, PdPt, PtRh, RhAg, RhIr, and RuPt are selected as promising candidates that might deviate from the scaling relations between O and OH. We note that the performance of the SG rules can be systematically improved by retraining with more data, for instance including information on alloys.

Overall, our results demonstrate the potential of SGD to detect complex local patterns associated to outstanding behavior. In particular, we showed here how the SGD approach can be applied to identify rules describing statistically exceptional data points associated (i) to a specific (range of) desired value(s) of a target property and (ii) to the largest deviations from a given model. Furthermore, generalizable SG rules were derived based on extremely small data sets compared to those typically needed for widely-used artificial-intelligence methods. This makes the SGD approach useful for several catalysis and materials-science applications in which only small (consistent) data sets are available. This contribution also demonstrates how the sharing of well-annotated FAIR (Findable, Accessible, Interoperable, and Re-purposable) data, increasingly available via common data infrastructures [31], can enable scientific insights beyond the original purpose for which the data was created and used.

Even though the SGD approach enables the screening of new materials, as demonstrated above, it does not provide predictions of oxygen adsorption energies for each different adsorption site. In particular, the SGD rule might not indicate the most stable surface sites for a given surface containing several possible adsorption sites on which oxygen might bind with different strength. However, knowing the relative stability of adsorption configurations might be important for the description of a catalytic process. This is addressed in reference [8] by using the sure-independence-screening-and-sparsifying-operator approach [32]. Similarly, we note that other AI strategies have been developed and applied for the accurate estimation of adsorption energies [33, 34]. Contrary to such global approaches, however, SGD provides a local description focused only on specific desired behaviors. Furthermore, SGD identifies simple constraints on the most relevant input parameters, which are helpful for rationalizing the possible underlying phenomena. The SGD analysis presented here thus advances the physical understanding of the *local* behavior with respect to global modelling approaches. Finally, we note that the dynamic restructuring of the catalyst material that might occur under reaction conditions, influencing the surface structure on which the reactions take place [1, 2], is not being taken into account in our analysis. This requires alternative modelling strategies [3, 35, 36].

## Comparison of Subgroup Discovery with Decision-Tree Regression

We also trained regression trees (RTs) [37] using the same data sets of targets and descriptive parameters as for SGD (see details in ESI). Similar to SGD, RTs also provide rules describing subsets of data identified during the training. These subsets of data are called “leaves”, and RTs provide predictions for the values of the target according to the leaf to which a given data point belongs.

For the ${\Delta }^{\mathrm{O}}$ target, the RT approach identified, on the leaf with the minimum predicted value of 0.12 eV, adsorption sites of Ag (fcc (111), fcc-s (211), fcc-t (211), and hcp-t (211)) and Pt (hollow (100), fcc-t (211), hcp-t (211)) metals. In total, 7 adsorption sites were selected. The rules for this leaf are:

13 $$ \begin{aligned}   \sigma ^{{{\text{O,RT}}}}  &  \equiv \varepsilon _{d}  \le  - 1.387~{\text{eV}} \\     &  \wedge ~{\text{site}}_{{{\text{nnd}}}}  \ge 2.651~{\text{\AA}} \\     &  \wedge {\text{IP}} \le 9.04~{\text{eV}} \\     &  \wedge f_{{sp}}  \ge 1.109 \\     &  \wedge {\text{DOS}}_{d}  \le 1.71~{\text{eV}}^{{ - 1}} . \\  \end{aligned}  $$

We applied the RT rule (13) to the test set of alloys. The selected surface sites are shown as orange crosses in Fig. 3A and B. The RT rule selects several of the alloys systems for which the DFT-calculated ${\Delta }^{\mathrm{O}}$ is relatively low. However, the distribution of ${\Delta }^{\mathrm{O}}$ values within the surface sites selected by the RT rule (orange bars in Fig. 3B) is broader than the corresponding distribution within the surface sites selected by the SG rule (black bars in Fig. 3B). Furthermore, the RT rule misses several relevant sites, including the fcc-t site of the AgPd alloy, for which the calculated ${\Delta }^{\mathrm{O}}$ is equal to zero. Such site is correctly selected by the SG rule. These results indicate that the RT rule is less focused on the outstanding sites compared to the SG rule.

For the ${\Delta }_{\mathrm{scaling}}^{\mathrm{O},\mathrm{OH}}$ target, the RT approach identifies, in the leaf with maximum predicted value of 0.42 eV, 6 adsorption sites. The rules describing this leaf are:

14 $$ \begin{aligned}   \sigma ^{{{\text{O}},{\text{OH}},{\text{RT}}}} &  \equiv \varepsilon _{d}  \le  - 1.805~{\text{eV}} \\ &  \wedge {\text{PE}} \le 2.41 \\ &  \wedge {\text{EA}} \ge 1.27~{\text{eV}} \\     &  \wedge {\text{CN}} \ge 7.667. \\  \end{aligned}  $$

Interestingly, (14) and the SG rule (12) select the exact same subset of training data. When applied to the test set of alloys (Fig. 5A and B, in orange), the RT rule also selects similar alloy surface sites compared to (12). However, it misses the IrRu fcc-s-1 (211) site (presenting ${\Delta }_{\mathrm{scaling}}^{\mathrm{O},\mathrm{OH}}=0.25 \mathrm{eV}$), which is correctly selected by (12) as a surface site that deviates from the linear-scaling relation. In spite of selecting similar training and test data, (12) and (14) provide significantly different results when applied to the exploitation set of alloys (Fig. 5C). In particular, the RT rule indicates that some bridge sites (for which ${\mathrm{site}}_{\mathrm{no}}=2$) could break the scaling relations, while this is not the case for the sites selected by the SG rule nor for the training set (Fig. 4A).

We ascribe the worse performance of the RT approach with respect to SGD for the present data set to the global character of the loss function used to select the rules in RT. Indeed, the loss function minimized during the training is, for RT, the prediction error over the *entire* data set. The few statistically exceptional cases therefore do not significantly impact the choice of rules. In SGD, in contrast, the rule is dictated mostly by the exceptional data points.

While the decision-tree approach can be used in combination with a categorical target as a *classifier* rather than a *regressor*, which allows for a more focused loss function, such strategy requires that the thresholds used for classification are specified a priori. For the case of the ${\Delta }^{\mathrm{O}}$ target discussed in Figs. 2 and 3, the extremes of the [1.30, 2.30 eV] interval used to define the target for SGD in (5) could be used as the classification thresholds (see results in ESI). However, for the general case of identifying rules for data points associated to a specific target value or for data points which deviate the most from a given model (as for the ${\Delta }_{\mathrm{scaling}}^{\mathrm{O},\mathrm{OH}}$ target discussed in Figs. 4 and 5), the choice of thresholds for a decision-tree classification approach, which can impact the resulting rules, might be nontrivial. This information is not required as input for the SGD approach.

## Conclusions

In this paper, we applied the SGD approach to identify the most relevant atomic, bulk and surface properties—as well as rules associated to those parameters—describing outstanding SGs of transition-metal surface sites. In particular, we demonstrated this approach using a data set of DFT-calculated adsorption energies [8, 24] by searching for surface sites (i) that present optimal range of oxygen binding strength for the ORR or (ii) that deviate the most from the linear-scaling relations between O and OH adsorption energies that impose a limit to the OER performance. The SGs rules not only hint at the relevant underlying physicochemical processes that govern the local statistically exceptional behavior, but are also suitable for guiding the design of challenging bimetallic alloys.

## Supplementary Information

Additional SGD and decision tree details are available as ESI. The SGD analysis described in this publication can be found in a Jupyter notebook at the *NOMAD Artificial-Intelligence Toolkit* (https://nomad-lab.eu/AItutorials/), where it can be repeated and modified directly in a web browser.. Below is the link to the electronic supplementary material.

Supplementary file1 (DOCX 3792 kb)

## Data Availability

All data analyzed in this study are included in this published article as supplementary information files. The SGD analysis described in this publication can be found in a Jupyter notebook at the NOMAD Artificial-Intelligence Toolkit (https://nomad-lab.eu/AItoolkit/sgd_alloys_oxygen_reduction_evolution), where it can be repeated and modified directly in a web browser.

## Code Availability

The SGD analysis presented in this paper was performed with CREEDO, a web application that provides an intuitive graphical user interface for real knowledge discovery algorithms and allows to rapidly design, deploy, and conduct user studies. CREEDO is available under http://realkd.org/creedo-webapp/. See also the NOMAD analytics-toolkit for a tutorial.
