# Supplementary material for: Identifying Outstanding Transition-Metal-Alloy Heterogeneous Catalysts for the Oxygen Reduction and Evolution Reactions via Subgroup Discovery
Source: Top Catal. 2021 Sep 2;65(1-4):196–206. doi: 10.1007/s11244-021-01502-4 (PMC8816773; doi:10.1007/s11244-021-01502-4)
Supplement: Supplementary file 1 — Supplementary file1 (DOCX 3792 kb) [file 11244_2021_1502_MOESM1_ESM.docx]

**Electronic Supplementary Information (ESI)**

Identifying outstanding transition-metal-alloy heterogeneous catalysts for the oxygen reduction and evolution reactions via subgroup discovery

Lucas Foppa* and Luca M. Ghiringhelli

The NOMAD Laboratory, Fritz-Haber-Institut der Max-Planck-Gesellschaft, Faradayweg 4-6, D-14195 Berlin, Germany; Humboldt-Universität zu Berlin, Zum Großen Windkanal 6, D-12489 Berlin, Germany.

*foppa@fhi-berlin.mpg.de

**Additional subgroup discovery details**

We performed the SGD analysis using the CREEDO code version 0.5.1.^1^ The cut-off values ($v_{j}$ in Eq. 3) used in the propositions were determined by k-means clustering using 10 clusters. We performed the SGD analysis using slightly different values of k (e.g. 8, 12) and verified that the similar SGs maximizing the quality-function value are obtained, i.e., SGs with similar rules which select the exact same subsets of data. The “randomized exceptional subgroup discovery” algorithm based on a Monte Carlo search was used for the SG search. Due to the stochastic nature of the search algorithm, we ran the SG search multiple times and analyzed the SGs obtained in different runs. We highlight that even though the list of SGs ranked according to the quality-function values changes every time the search algorithm is executed, the quality-function values for a fixed SG always assume the same value. We focus the discussion on the SGs that provide the maximum quality-function value. For the case of the $\Delta^{O}$ target, we further looked among the SGs identified with near-optimal quality function and selected the SGs presenting the highest utility-function value (labelled as SG^O^, in black in Fig. S1) for discussion.

By using the function defined in Eq. 5, we allow for SGD to focus on a range of desired target values based on the distance to the optimal value. Another strategy to achieve this goal is to define a categorical target which labels the data points falling in the desired range of values. In this case, the data points inside or outside the desired range are treated equivalently irrespective of their distance to the optimal value or to the borders delimiting the range. To illustrate this approach, we have applied SGD using a categorical target indicating whether a surface site is at the optimal [1.30, 2.30 eV] oxygen adsorption energy range:

$\delta_{\varepsilon}^{O}=\left\{ \begin{aligned} 1, &{{(E}_{ads,opt}^{O}-\varepsilon)\leq E}_{\mathrm{ads}}^{O}\leq{(E}_{ads,opt}^{O}+\varepsilon) \\ 0, &\mathrm{otherwise} \end{aligned} \right.$, (S1)

where$\varepsilon$ determines the range of oxygen adsorption energy values considered optimal. Thus, the SGD is used to identify rules that *classify* the adsorption sites and materials as belonging to the Sabatier-optimal range. By using this approach, SGD identified similar rules compared to those derived using the numerical target $\Delta^{O}$. Additionally, the same subselection of data points as for the SG discussed in Fig. 2 is obtained. This is also the case when the optimal ranges of [1.40, 2.20 eV] or [1.20, 2.40 eV] are used, instead of [1.30, 2.30 eV], for the labelling of data points. The SGs identified for each of these values are shown in Table S1. These results indicate that the SG rules are stable with respect to the choice of target function and optimal range of values around the proposed optimum (within the ranges $\pm$0.4-0.6 eV).

For the SGD approach with this categorical target, the total variation distance, denoted $D_{\mathrm{TV}}$, between the SG and the whole data set is used as utility function. $D_{\mathrm{TV}}$ measures the largest possible difference between the probabilities that two probability distributions $P$ and $P$‘ can assign to the same event. $D_{\mathrm{TV}}$ can be defined, for discrete distributions, as

$D_{\mathrm{TV}}\left( P,P^{'} \right)=\frac{1}{2}{\left\| P \right.\left. -P^{'} \right\|}_{1}=\frac{1}{2}\sum_{x\epsilon\chi} |P\left( x \right)-P^{'}\left( x \right)|$, (S2)

where ${||P-P'||}_{1}$ denotes the L1 norm and $x$ runs over the probability space $\chi$. We note that the class of interest is not specified in the SGD approach with the categorical target. However, we do verify that the identified SGs correspond to $\delta_{\varepsilon}^{O}=1$, i.e., to the materials and adsorption sites associated to the desired Sabatier-optimal range.

**Jensen-Shannon divergence in subgroup discovery**

In our SGD analysis of SGs of surface sites that deviate from the scaling relations, we use $D_{\mathrm{cJS}}$ the cumulative-distribution function formulation of $D_{\mathrm{JS}}$, the Jensen-Shannon divergence,to quantify, along with the coverage term, how outstanding a SG is. $D_{\mathrm{JS}}$ is a measure of dissimilarity between two distributions (e.g., $P$ and $P'$). It is defined by

$D_{\mathrm{JS}}(P||P')=\frac{1}{2}D_{\mathrm{KL}}(P|\left| M \right)+\frac{1}{2}D_{\mathrm{KL}}(P'|\left| M \right)$ , (S3)

where $M=\frac{P+P'}{2}$ and $D_{\mathrm{KL}}$ is the Kulback-Leibler divergence. $D_{\mathrm{JS}}$ is a symmetrized version of $D_{\mathrm{KL}}$, i.e., the same divergence is obtained irrespective of the choice of $P$ and $P'$. $D_{\mathrm{KL}}$ is defined, in the case of discrete distributions, by

$D_{\mathrm{KL}}(P||P')=\sum_{x\epsilon\chi} P\left( x \right)\log\frac{P(x)}{P'(x)}$ , (S4)

where $\chi$ indicates the probability space. $D_{\mathrm{KL}}$ is also called relative entropy, due to the similarity of its expression with the Shannon entropy for a random variable$X$:

$H\left( X \right)=-\sum_{x\epsilon\chi} P\left( x \right)logP\left( x \right) .$(S5)

In order to get an intuition on how the $D_{\mathrm{JS}}$ influences the SGD approach, we evaluated the $D_{\mathrm{JS}}$ between a fixed normal distribution $P$ (shown in orange in Fig. S2) and several normal distributions $P'$ (shown in black in Fig. S2) whose mean value or narrowness were modified with respect to the $P$. In the context of SGD, $P$ can be seen as the distribution of the target over the whole data set, whereas$P'$ is analogous to the several possible SGs of the data set. We note that the narrowness corresponds to the standard deviation of the distributions. Fig. S2 shows that when $P$ and $P'$ are the exact same distribution, $D_{\mathrm{JS}}$ is equal to zero. However, the more $P'$ mean value is shifted with respect to $P$, the highest the $D_{\mathrm{JS}}$ gets (Fig S2, horizontal panels). Similarly, the narrower $P'$ is with respect to $P$, the highest the $D_{\mathrm{JS}}$ gets (Fig S2, vertical panels). Because the quality function is *maximized* during the SG search, the more shifted and narrow the SG is, the more outstanding it will be considered. Finally, we note that $D_{\mathrm{cJS}}$ is used in our SGD approach because it can be efficiently calculated from the data without the need for estimations.

**Decision tree approach**

Prior to the training of the regression tree models, we determined the appropriate maximum tree depth ($\mathrm{tree}_{\mathrm{depth}}$) and minimum number of samples in a split ($\mathrm{tree}_{\mathrm{split}}$), i.e., the regression-tree hyperparameters, using cross-validation. For this purpose, we performed leave-10%-out cross validation, i.e., we trained regression trees at the different hyperparameter values using 90% of the data and evaluated test errors on the left-out 10% of the data. The selections of train and test subsets were performed randomly, using 500 independent selections. We selected the hyperparameters associated to the minimum test root-mean-squared error (RMSE) averaged over all cross-validation iterations. For the target $\Delta^{O}$ (Fig. S4), the identified $\mathrm{tree}_{\mathrm{depth}}$ and $\mathrm{tree}_{\mathrm{split}}$ are 1 and 2, respectively. For the target $\Delta_{\mathrm{scaling}}^{O,OH}$ (Fig. S7), two minima are identified, corresponding to $\mathrm{tree}_{\mathrm{depth}}$ and $\mathrm{tree}_{\mathrm{split}}$ of 4 and 2; or 9 and 4, respectively. We focus the discussion on the model obtained with a lower $\mathrm{tree}_{\mathrm{depth}}$ value ($\mathrm{tree}_{\mathrm{depth}}$ and $\mathrm{tree}_{\mathrm{split}}$ of 4 and 2), which gives simpler, and thus in principle more generalizable, rules - and predictions. The *sklearn* library was used.

We have also trained a decision tree classifier using the categorical target defined in S1 with $\varepsilon=0.50 \mathrm{eV}$. The Gini impurity was used to measure the quality of the splits. The model hyperparameters $\mathrm{tree}_{\mathrm{depth}}$ and $\mathrm{tree}_{\mathrm{split}}$ were determined via cross-validation analogously to the case of the regression tree, i.e. using 500 iterations of leave-10%-out cross validation. We used the the classification score as metric to choose the hyperparameters (Fig. S5A). The identified optimal $\mathrm{tree}_{\mathrm{depth}}$ and $\mathrm{tree}_{\mathrm{split}}$ are both equal to 2. The rules defining the surface sites with oxygen adsorption energy in the range [1.30, 2.30 eV] given by the model trained with the whole data at the optimal hyperparameters are

$\mathrm{site}_{\mathrm{nnd}}>2.782 Å\wedge EA\leq2.217$. (S6)

These rules correctly select 17 out of 18 surface sites belonging to the desired range of oxygen adsorption energy (Fig. S5B). Moreover, 4 surface sites outside the desired range are incorrectly selected by (S6). The performance of the rules defined by (S6) for selecting the outstanding alloy surface sites of the test set is shown in in Fig. S6A and S6B. We observe that the rule obtained with the decision tree classification approach selects more of the relevant adsorption sites with low $\Delta^{O}$ compared to the regression tree approach (Fig. 3A and 3B). However, more data points are selected with the classification compared to the regression approach (19 vs. 9 data points, respectively) and the selection presents a broader distribution of $\Delta^{O}$ values for the classification vs. the regression tree rule. This indicates that the classification approach provides less focused rules.

**Table S1.** SGs identified by SGD that present different rules but correspond to the same subselection of data points. The SGs shown in bold are those discussed in the main text.

| target | $u\left( P,SG \right)$ | $s(SG)$ | $u\left( P,SG \right)$ | selectors |
| --- | --- | --- | --- | --- |
| $\Delta^{O}$ | $\frac{std(SG)}{std(P)}$ | 23 | 0.919 | $\boldsymbol{2.786<}\mathbf{bulk}_{\mathbf{nnd}}\boldsymbol{\leq}\boldsymbol{2.987 Å}$ |
|  |  |  |  | $\mathbf{site}_{\mathbf{nnd}}\boldsymbol{>2.759 Å\wedge}\boldsymbol{PE\leq2.125}$ |
|  |  |  |  | $7.518\leq\mathrm{IP}\leq8.959 \mathrm{eV}\wedge V_{\mathrm{ad}}^{2}\geq2.26$ |
|  |  |  |  | $r_{d}\geq0.8 Å\wedge\mathrm{IP}\leq8.959 \mathrm{eV}\wedge f_{d}\geq8.326$ |
|  |  |  |  | $\mathrm{bulk}_{\mathrm{nnd}}>2.744 Å\wedge IP\leq8.959$ |
|  |  |  |  | $\mathrm{bulk}_{\mathrm{nnd}}\leq2.987 Å\wedge r_{d}\geq0.78 Å\wedge f_{d}\geq8.732$ |
| $\delta_{0.4}^{O}$ | $D_{\mathrm{TV}}(P,SG)$ | 23 | 0.484 | $\mathrm{bulk}_{\mathrm{nnd}}>2.786 Å\wedge PE\leq2.28$ |
|  |  |  |  | $\mathrm{IP}\leq9.121 \mathrm{eV}\wedge f_{d}>8.732\wedge PE\geq1.92$ |
|  |  |  |  | $\mathrm{site}_{\mathrm{nnd}}>2.759 Å\wedge PE\leq2.28$ |
|  |  |  |  | $\mathrm{bulk}_{\mathrm{nnd}}>2.786 Å\wedge IP\leq9.121 eV$ |
|  |  |  |  | $\mathrm{bulk}_{\mathrm{nnd}}>2.786 Å\wedge EA\leq2.125 eV$ |
|  |  |  |  | $\mathrm{site}_{\mathrm{nnd}}\geq2.633 Å\wedge EA\leq2.125 eV\wedge f_{d}\geq8.326$ |
| $\delta_{0.5}^{O}$ | $D_{\mathrm{TV}}(P,SG)$ | 23 | 0.550 | $\mathrm{bulk}_{\mathrm{nnd}}>2.786 Å\wedge PE\leq2.28$ |
|  |  |  |  | $\mathrm{site}_{\mathrm{nnd}}\geq2.633 Å\wedge IP\leq8.959 eV\wedge f_{d}\geq8.326$ |
|  |  |  |  | $V_{\mathrm{ad}}^{2}\geq1.59\wedge EA\leq2.125 eV\wedge f_{d}>8.732$ |
|  |  |  |  | $\mathrm{site}_{\mathrm{nnd}}>2.708 Å\wedge PE\leq2.28\wedge f_{d}>8.732$ |
|  |  |  |  | $\mathrm{bulk}_{\mathrm{nnd}}\geq2.733 Å\wedge IP\leq9.121 eV\wedge f_{d}>8.732$ |
|  |  |  |  | $r_{d}\geq0.89 Å\wedge\mathrm{IP}\leq8.959\wedge f_{d}\geq8.326$ |
| $\delta_{0.6}^{O}$ | $D_{\mathrm{TV}}(P,SG)$ | 23 | 0.681 | $\mathrm{bulk}_{\mathrm{nnd}}>2.786 Å\wedge PE\leq2.28$ |
|  |  |  |  | $\mathrm{site}_{\mathrm{nnd}}>2.759 Å\wedge PE\leq2.28$ |
|  |  |  |  | $V_{\mathrm{ad}}^{2}\geq1.34\wedge EA\leq2.125 eV\wedge f_{d}\geq8.326$ |
|  |  |  |  | $2.744< \mathrm{bulk}_{\mathrm{nnd}}\leq2.987 Å\wedge f_{d}\geq7.829$ |
|  |  |  |  | $r_{d}\geq0.89 Å\wedge7.518\leq\mathrm{IP}\leq8.959 \mathrm{eV}$ |
|  |  |  |  | $\mathrm{bulk}_{\mathrm{nnd}}>2.786 Å\wedge IP\leq9.121 eV$ |
| $\Delta_{\mathrm{scaling}}^{O,OH}$ | $D_{\mathrm{cJS}}(P,SG)$ | 6 | 0.457 | $\mathbf{site}_{\mathbf{no}}\boldsymbol{>2.5\wedge}\boldsymbol{1.236<EA\leq2.125} \mathbf{eV}$ |
|  |  |  |  | $CN\geq7.667\wedge EA>1.236 eV \wedge\mathrm{PE}\boldsymbol{\leq}2.28$ |
|  |  |  |  | $\mathrm{site}_{\mathrm{nnd}}\geq2.638 Å\wedge CN\geq8.333 \wedge EA\geq1.15 eV\boldsymbol{\wedge}PE\leq2.28$ |
|  |  |  |  | $\mathrm{site}_{\mathrm{no}}\geq3\wedge\epsilon_{d}<-1.805 eV\boldsymbol{\wedge}1.92\leq PE\leq2.28$ |
|  |  |  |  | $\mathrm{site}_{\mathrm{no}}\geq3\wedge\mathrm{IP}\leq9.121 eV\wedge EA\geq1.15 eV\boldsymbol{\wedge}PE\geq1.92$ |
|  |  |  |  | $V_{\mathrm{ad}}^{2}\geq1.34\wedge\mathrm{CN}\geq8.333\wedge\epsilon_{d}<-1.805 eV\boldsymbol{\wedge}PE\leq2.28$ |

**Figure S1.** SGs with near-optimal quality function identified for the target $\Delta^{O}$. The SG^max^ (in blue) is the SG with the highest quality-function value. The SG^O^ (in black) is the SG with the highest value of utility function ($\frac{std(SG)}{std(P)}$) among the SGs that have high quality-function values (in grey). SG^O^ is the SG discussed in the main text.

**Figure S2.** Illustration of the Jensen-Shannon divergence. (Horizontal panels) $D_{\mathrm{JS}}$ between normal distributions with the same narrowness but different mean values. (Vertical panels) $D_{\mathrm{JS}}$ between normal distributions with the same mean values but different narrowness. The narrowness corresponds to the standard deviation of the distributions.

**Figure S3.** Pearson correlation between the candidate descriptive parameters. The color scale indicates the values of the correlation score, which are explicitly indicated in the graph.

**Figure S4.** Regression tree for the target $\Delta^{O}$. A: Cross-validation analysis for hyperparameter optimization. B: Regression tree trained with the whole data set of monometallic systems at the identified optimal hyperparameter values ($\mathrm{tree}_{\mathrm{depth}}=5$, $\mathrm{tree}_{\mathrm{split}}=3$, indicated in A). The leaf in white corresponds to the surface sites of interest, presenting the lowest predicted target value of 0.115 eV.

**Figure S5.** Classification tree for the target $\Delta^{O}$. A: Cross-validation analysis for hyperparameter optimization. B: Classification tree trained with the whole data set of monometallic systems at the identified optimal hyperparameter values ($\mathrm{tree}_{\mathrm{depth}}=2$, $\mathrm{tree}_{\mathrm{split}}=2$, indicated in A). The leaf in orange corresponds to the surface sites of interest, presenting the oxygen adsorption energy value in the range [1.30, 2.30 eV].

**Figure S6.** SG rules for monometallic surface sites with optimal range of oxygen adsorption energies applied for the design bimetallic alloys. A: Representation of the test set of alloy surface sites in the coordinates of the key descriptive parameters identified in the SG rule (8): $\mathrm{site}_{\mathrm{nnd}}$ and $\mathrm{PE}$. The data points are colored according to their $\Delta^{O}$ value. The data points selected by the SG rule (8) and by the classification (class.) tree rule (S6) are shown in black and red crosses, respectively. B: Distribution of $\Delta^{O}$ values in the test set of alloy surface sites (grey). The distributions of $\Delta^{O}$ values over the data points selected by the SG rule (8) and the classification tree rule (S6) are displayed in black and red, respectively. C: Representation of the exploitation set of alloy surface sites in the coordinates $\mathrm{site}_{\mathrm{nnd}}$ and $\mathrm{PE}$. The data points selected by the SG rules shown in Table S1 (for the $\Delta^{O}$ target) and by the classification tree rule (S6) are shown in black and red crosses, respectively.

**Figure S7.** Regression tree for the target $\Delta_{\mathrm{scaling}}^{O,OH}.$ A: Cross-validation analysis for hyperparameter optimization. B: Regression tree trained with the whole data set of monometallic systems at the identified optimal hyperparameter values ($\mathrm{tree}_{\mathrm{depth}}=4$, $\mathrm{tree}_{\mathrm{split}}=2$, indicated in A). The leaf with the darkest color corresponds to the surface sites of interest, presenting the highest predicted target value of 0.418 eV.

**Reference**

1. CREEDO is a web application that provides an intuitive graphical user interface for real knowledge discovery algorithms and allows to rapidly design, deploy, and conduct user studies. See http://realkd.org/creedo-webapp/for additional information. See also the NOMAD analytics-toolkit for a tutorial.
